# Left atrial strain reveals subclinical dysfunction in children after successful coarctation repair

**DOI:** 10.1371/journal.pone.0344778

**Published:** 2026-03-11

**Authors:** Thuy Thuc Minh Pham, Phuc Nang Vu, Chi Thi Khanh Nguyen, Hung Quoc Nguyen, Thu-Tinh Nguyen, Phuc Minh Vu

**Affiliations:** 1 School of Medicine, University of Medicine and Pharmacy at Ho Chi Minh City, Ho Chi Minh City, Vietnam; 2 Cardiovascular Center, Tam Anh General Hospital, Ho Chi Minh City, Vietnam; 3 Department of Pediatric Cardiology, Ha Noi Heart Hospital, Hanoi, Vietnam; 4 Department of Cardiology, Children’s Hospital 1, Ho Chi Minh City, Vietnam; Siloam Hospitals Lippo Village, INDONESIA

## Abstract

**Background:**

Although surgical repair of aortic coarctation (CoA) often restores hemodynamic integrity, subclinical myocardial dysfunction may persist long-term. Left atrial (LA) strain is a sensitive marker of atrial compliance and early diastolic dysfunction, but its role in pediatric CoA is underexplored.

**Methods:**

The study was a multicenter, cross-sectional, observational study conducted at three tertiary hospitals in Vietnam. We enrolled 34 children with isolated CoA who underwent successful surgical repair, recruited between December 2022 and March 2025. LA volumes and strain, with LA reservoir strain (LASr) as the primary parameter, were assessed by two-dimensional speckle-tracking echocardiography. Values were compared with published pediatric reference data. Left ventricular ejection fraction (LVEF) and global longitudinal strain (GLS) were also measured.

**Results:**

Despite preserved LVEF and normal anatomy, mean LASr was significantly reduced in the CoA group (37.6 ± 6.2%) compared to reference values (47.3%; p < 0.001). LAScd was also significantly reduced (23.4 ± 8.3%) compared to reference values (32.8%, p < 0.001). LA dilation was present in 18–56% of patients, but LASr was consistently impaired. Importantly, LASr did not significantly correlate with LVEF, GLS, blood pressure, or LA volume, suggesting it identifies an independent subclinical atrial dysfunction. Children with stage 1 hypertension had significantly lower LASr and larger LA volume.

**Conclusions:**

Subclinical left atrial dysfunction is common in children after successful CoA repair, even in the absence of residual obstruction or systolic impairment. Measuring LA strain may provide incremental value for early risk stratification and long-term follow-up in this population.

## Introduction

Coarctation of the aorta (CoA) is a congenital narrowing of the thoracic aorta that increases left ventricular (LV) afterload. It contributes to long-term cardiovascular morbidity, even after successful surgical or catheter-based repair [1–3]. While anatomical correction typically resolves the obstruction, many patients continue to exhibit residual hypertension, vascular stiffness, or subclinical cardiac dysfunction during follow-up [4–6].

Recent research has highlighted the role of left atrial (LA) function as an early marker of cardiovascular risk. LA remodeling and dysfunction have been associated with diastolic dysfunction, elevated filling pressures, and adverse outcomes in both pediatric and adult populations [7–9]. Among echocardiographic parameters, LA reservoir strain assessed by speckle-tracking echocardiography (STE) is a validated and reproducible measure of atrial compliance and diastolic performance [10,11].

In adults with repaired CoA, impaired LA strain has been linked to increased risk of arrhythmia and diastolic dysfunction, independent of LV systolic function [12]. However, studies on LA function post-CoA repair in children are scarce. Most pediatric research focuses on ventricular performance [10,13–15] and normative data for LA strain in children remain limited [11]. Consequently, early atrial dysfunction may go undetected in routine pediatric care.

This study aimed to assess LA size and phasic strain using two-dimensional STE in children after anatomically successful CoA repair. We hypothesized that LA reservoir strain would be impaired despite preserved LV systolic function and normal arch anatomy, and that LA strain could serve as an early marker of subclinical atrial dysfunction in this population.These findings may support the incorporation of the LA strain into routine long-term surveillance of children after CoA repair.

## Materials and methods

### Study design and population

We conducted a multicenter cross-sectional observational study involving 34 children (aged under 18 years) with isolated coarctation of the aorta who were followed at three tertiary centers. This multicenter study recruited participants from Tam Anh General Hospital (15 December 2022–31 December 2025, data collection ongoing), Children’s Hospital 1 (01 March 2023–31 December 2025, data collection ongoing), and Hanoi Heart Hospital (20 June 2024–30 June 2025). For this analysis, we included all participants who met the inclusion criteria and were recruited up to 1 March 2025. All patients had undergone surgical repair via end-to-end anastomosis at least six months before enrollment and fulfilled predefined criteria for successful correction, including a mean Doppler gradient less than 20 mmHg across the aortic isthmus and no systolic blood pressure discrepancy between the upper and lower limbs. Patients with additional congenital heart defects (other than bicuspid aortic valve or ligated patent ductus arteriosus), moderate or greater valvular disease, or implanted pacemakers were excluded.

The study was conducted in accordance with the Declaration of Helsinki, with ethics approval obtained from the relevant institutional review boards, and written informed consent secured from all participants’ parents or legal guardians.

### Blood pressure measurement

Blood pressure was measured in all four limbs after at least five minutes of rest in the supine position, using an automated oscillometric device. For classification purposes, the highest systolic pressure recorded from the upper limbs was used. Hypertension was defined based on the 2017 guidelines of the American Academy of Pediatrics [7].

### Echocardiographic acquisition and analysis

Transthoracic echocardiography was performed using GE Vivid E95, IQ, and S70N systems. Standard pediatric imaging protocols were followed in accordance with the American Society of Echocardiography recommendations [8].

**Fig 1:**
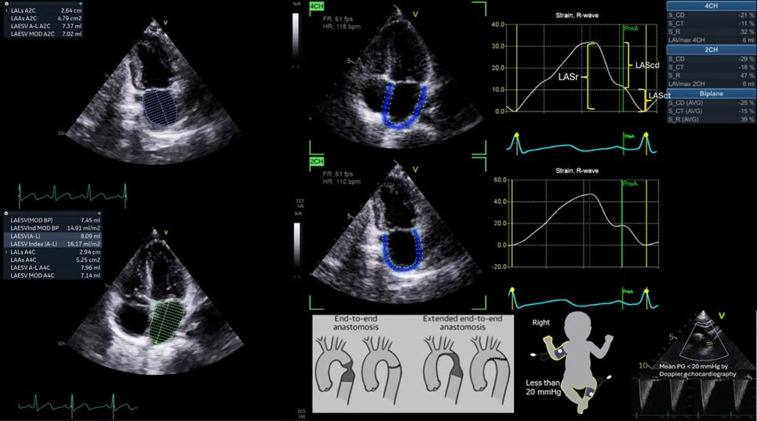
Representative left atrial phasic strain analysis by speckle-tracking echocardiography in a child post-coarctation repair. Apical four-chamber (top) and two-chamber (bottom) views demonstrate LA endocardial tracking and corresponding strain curves aligned to the R-wave. Phasic strain components: reservoir (S-R), conduit (S-CD), and contraction (S-CT) were derived using biplane assessment.

Left atrial volumes were measured at ventricular end-systole from apical four-chamber and biplane (area–length) views (Fig 1) and indexed to body surface area (BSA) using the Haycock formula. Pediatric Z-scores were calculated using established reference datasets [11,15] Left atrial strain was assessed using two-dimensional speckle-tracking echocardiography on apical four-chamber and two-chamber views, with frame rates between 50 and 90 frames per second. Imaging depth and sector width were adjusted to optimize temporal and spatial resolution. All acquisitions were performed by a single experienced investigator (PTTM) following a standardized protocol across all study sites. Offline analysis was conducted using *EchoPAC Software Only version 206 (*GE Healthcare*, Chicago, IL, USA*) (Fig 1). Left atrial endocardial borders were manually traced at end-systole, and a region of interest (ROI) was automatically generated and adjusted to cover the LA myocardium. The software tracked atrial myocardial motion during the cardiac cycle and generated corresponding strain curves. Reservoir, conduit, and contraction strain values were obtained, with left atrial reservoir strain (LASr) designated as the primary parameter based on its validated reproducibility and clinical relevance. LASr values were compared with the published pediatric reference data (mean 47.3%, 95% CI: 42.5–52.1%) [11]. Inter- and intra-observer reproducibility of LASr was assessed in 10 randomly selected patients using intraclass correlation coefficients.

Additionally, we performed an exploratory multivariable linear regression analysis to identify potential independent predictors of LA reservoir strain. Variables included in the model were age, body surface area (BSA), time since surgery, and hypertension status. Statistical significance was set at p < 0.05.

To assess measurement reproducibility, interobserver variability of left atrial reservoir strain (LASr) was evaluated in a randomly selected subset of 10 patients. LASr measurements were independently performed by a second experienced investigator who was blinded to both clinical data and the primary observer’s measurements. Agreement between observers was quantified using an intraclass correlation coefficient (ICC) based on a two-way random-effects model for absolute agreement. Bland–Altman analysis (S1 Fig) was additionally performed to assess systematic bias and 95% limits of agreement between observers.

Left ventricular ejection fraction (EF) was calculated using the biplane Simpson method. Global longitudinal strain (GLS) was also assessed using speckle-tracking echocardiography.

### Statistical analysis

All statistical analyses were conducted using Stata version 17.0 (*StataCorp, USA*). Continuous variables were presented as mean ± standard deviation or median with interquartile range, and categorical variables as counts and percentages. Group comparisons were performed using t-test or Mann–Whitney U test, and χ² or Fisher’s exact test where appropriate. Correlations were assessed using Pearson or Spearman coefficients. A two-sided p-value less than 0.05 was considered statistically significant.

A post hoc power analysis was conducted to assess whether the sample size was sufficient for the primary comparison of LA reservoir strain. Using our cohort mean (37.6 ± 6.2%) and a published reference value (47.3%, 95% CI: 42.5–52.1%) [11], the standardized effect size was z = 3.63, yielding a two-sided p ≈ 0.00028 and an estimated power of approximately 0.95 (α = 0.05). The minimal detectable difference at 80% power under these assumptions was approximately 7.5%.

### Ethics approval and consent to participate

The study protocol was approved by the Ethics Committee of the University of Medicine and Pharmacy at Ho Chi Minh City (IRB No. 721/HĐĐĐ-ĐHYD, 6 October 2022), with an amendment on 16 May 2024 (IRB No. 653/HĐĐĐ-ĐHYD) to expand the study sites. Additional approvals were obtained from Children’s Hospital 1 (IRB No. 181/GCN-BVNĐ1, 24 February 2023) and Hanoi Heart Hospital (IRB No. 1910/QĐ-BYT, 18 June 2024). Tam Anh General Hospital approved data collection and sample acquisition on 13 December 2022 under the supervision of the University of Medicine and Pharmacy ethics board. Written informed consent was obtained from the parents or legal guardians of all participants.

## Results

### Baseline characteristics

A total of 34 children with successfully repaired CoA were included. The median age at echocardiographic evaluation was 50.0 months (IQR 19.0–101.0), and 70.6% were male. The median time since surgery was 19.5 months (IQR 11.2–62.2). Median body weight and height were 13.5 kg (IQR 9.5–24.0) and 99 cm (IQR 80.0–123.0), respectively. Mean body surface area (BSA) was 0.64 ± 0.18 m², and 85.3% of children had a BSA less than 1.0 m². A bicuspid aortic valve was present in 50%. Only one patient (2.9%) was receiving antihypertensive medication. At follow-up, 76.5% had elevated blood pressure, including 55.9% with stage 1 hypertension. (Table 1).

**Table 1:** Characteristics of the study population.

Variables	Total (n = 34)	Percent (%)
Age (month)*	50.0 (19.0–101.0)	
**Group Age**		
< 2 years	12	35.3
≥ 2 years	22	64.7
**Gender**		
Male	24	70.6
Female	10	29.4
Weight* (kg)	13.5 (9.5–24.0)	
Height* (cm)	99 (80.0–123.0)	
BSA* (m^**2**^)	(0.4–0.9)	
≤ 1m^2^	29	85.3
> 1m^2^	5	14.7
SBP* (mmHg)	105.0 (100.0–113.0)	
DBP** (mmHg)	61.8 ± 10.4 (42.0–87.0)	
**Medications**		
Yes	1	2.9
No	33	97.1
**Months after surgery**	19.0 (9.0–59.0)	
**Hypertension**		
Normal	8	23.5
Elevated	7	20.6
Stage1	19	55.9

* Median (IQR) ** Mean ± SD (min–max).

### Left ventricular function

All patients demonstrated preserved left ventricular systolic function. The mean LVEF was 71.5 ± 4.8%, and the mean GLS was –19.6 ± 1.7%, both falling within the published pediatric reference range. However, 21 patients (61.8%) had GLS values that were below the median or near the lower limit of normal for their age group, based on reference values reported in a recent meta-analysis of over 1400 healthy children [16]. These borderline GLS values may reflect physiological interindividual variability or low-normal strain rather than definite systolic dysfunction (Table 2).

**Table 2:** Left ventricular function.

Variables	Total (n = 34)	Percent (%)
LVEF** (%)	71.5 ± 4.8 (64.0–82.0)	
**Group LVEF**		
Normal	34	100.0
LVEF Simpson BiP** (%)	61.7 ± 2.4 (57.0–72.0)	
GLS** (%)	(−19.6) ± 1.7 ((−23.1)–(−16.5))	
**Group GLS**		
Borderline	21	61.8
Normal	13	38.2

* Median (IQR) ** Mean ± SD (min–max).

**Fig 2:**
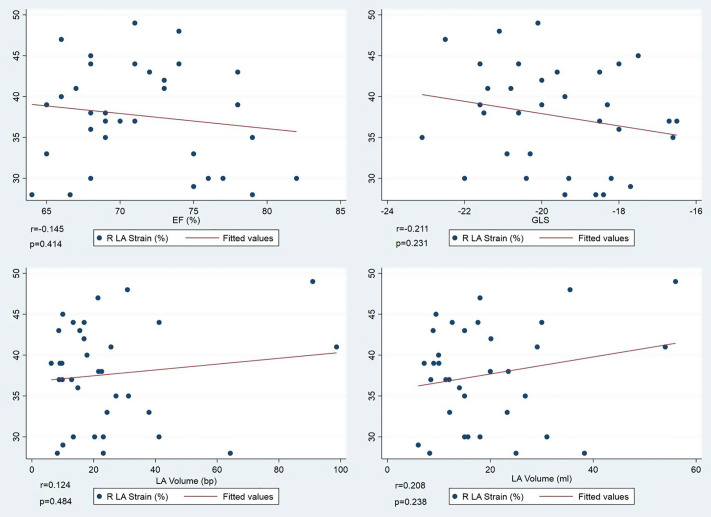
Correlation plots between left atrial (LA) reservoir strain and key echocardiographic parameters. No statistically significant correlations were observed in any of the comparisons. Top left: LA strain vs. left ventricular ejection fraction (LVEF) (r = –0.145, p = 0.414). Top right: LA strain vs. global longitudinal strain (GLS) (r = –0.211, p = 0.231). Bottom left: LA strain vs. LA volume indexed to body surface area (BSA) (r = 0.124, p = 0.484). Bottom right: LA strain vs. unindexed LA volume (r = 0.208, p = 0.238).

To further assess the relationship between LV and LA function, patients were stratified based on GLS values using a threshold of −18%, which approximates the lower limit of normal in some pediatric reference studies [13–15]. Among the 34 patients, 29 had GLS less than or equal to –18%, and 5 had GLS values greater than –18%. Children in the lower GLS group (n = 29) had significantly larger LA volumes as measured by the four-chamber method (median 18.0 [12.1–26.8] ml vs. 9.4 [8.4–11.4] mL, p = 0.012). However, no significant differences were observed in biplane LA volume (p = 0.308) or LA reservoir strain (p = 0.640) between the two groups (Fig 2).These results suggest a potential association between lower GLS values and increased LA size, although LASr appears to be relatively preserved regardless of minor variations in ventricular strain.

### Left atrial volume and strain

Left atrial volume and function were both altered in a substantial subset of children following CoA repair. LA dilation was observed in 17.6% of patients using four-chamber Z-scores and in 55.9% using the biplane area–length method, highlighting the variability across measurement techniques (Table 3).

**Table 3:** Left atrial volume and strain.

Variables	Total (n = 34)	Percent (%)
**LA vol (1) – apical 4 chamber view**		
LA ESV* (A4C) (ml)	15.3 (10.0–25.0)	
Z-Score	0.4 ± 1.6 ((−2.7)–5.5)	
Normal	28	82.3
Dilated	6	17.6
BSA_Haycock* (m^2^)	0.6 (0.5–0.9)	
≤ 1m^2^	29	85.3
> 1m^2^	5	14.7
**LA vol (2) – biplane area–length method**		
LA ESV* (bp) (ml)	19.1 (10.0–27.2)	
LA ESV index* (bp) (ml/m^2^)	28.2 (24.3–32.1)	
Dilated LA		
Normal	4	11.8
Borderline	11	32.3
Dilated	19	55.9
LASr** (%)	37.6 ± 6.2 (28.0–49.0)	
LAScd** (%)	23.4 ± 8.3 (1–38)	
LASct** (%)	12.5 (10 –17)	

* Median (IQR) ** Mean ± SD (min – max).

Echocardiographic acquisition was prospectively optimized according to standardized technical criteria for speckle-tracking analysis. Consequently, left atrial volumes and all strain parameters (LASr, LAScd, and LASct) were successfully analyzed in all 34 patients (100%), and no case was excluded from strain analysis due to inadequate image quality. Reproducibility analysis demonstrated excellent measurement reliability. In 10 randomly selected patients, interobserver agreement for LASr was excellent (ICC = 0.943), with narrow limits of agreement on Bland–Altman analysis (S1 Fig).

**Fig 3:**
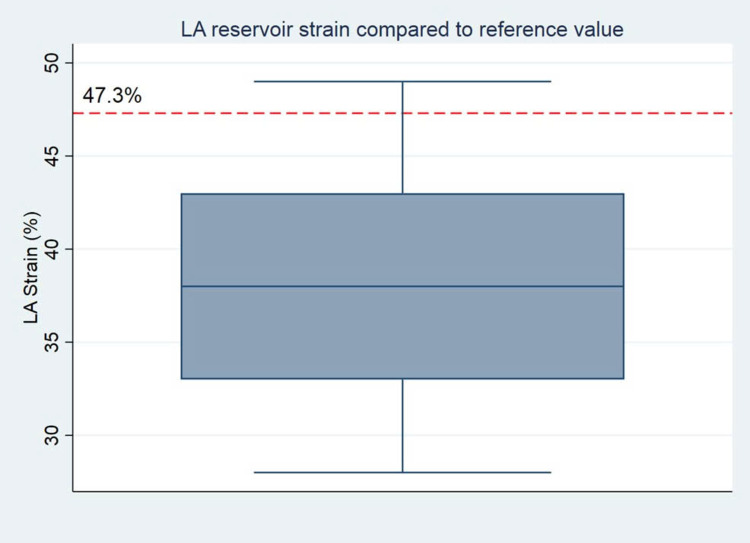
Boxplot of LA reservoir strain values in the study cohort. The dashed red line represents the reported pediatric reference value (47.3%). The majority of patients demonstrated reduced LA strain compared to the reference. These findings confirm that LA reservoir strain is frequently reduced in children after anatomically and hemodynamically successful CoA repair, even in the absence of LA dilation or impaired LV systolic function.

Although LA dilation was present in approximately 20% to 50% of patients, depending on the measurement method, LA reservoir strain was more consistently reduced. The mean value of 37.6 ± 6.2%, significantly lower than published pediatric reference values (mean 47.3%, 95% CI 42.5–52.1%; p < 0.001) (Fig 3). While these reductions suggest subclinical alterations, currently, there are no universally accepted pediatric cutoffs to define pathological LASr. Similarly, conduit strain (Scd) was significantly reduced compared to reference values (23.4 ± 8.3% vs. 32.8%, p < 0.001), while contractile strain (Sct) was slightly lower (14.4 ± 8.1% vs. 12.0%) but not statistically significant (p = 0.093). In continuous variable analysis, LA reservoir strain was not significantly correlated with LVEF, GLS, LA volume, or systolic blood pressure, suggesting that atrial dysfunction may precede structural remodeling or overt ventricular impairment (Fig 2).

### Subgroup analysis

**Fig 4:**
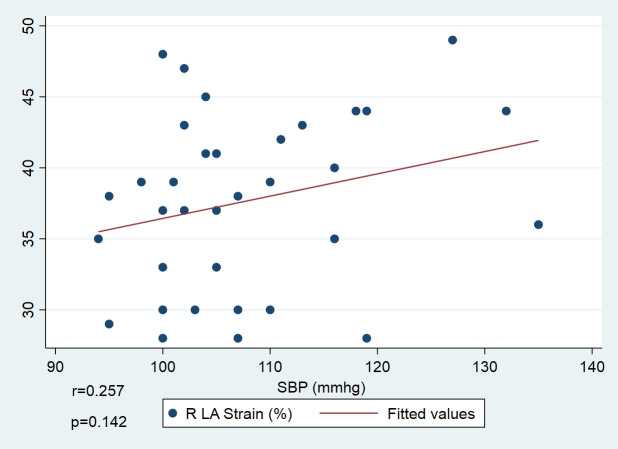
Comparison of left atrial volume and systolic blood pressure in children by left atrial strain status. Scatter plot showing the relationship between systolic blood pressure (SBP) and left atrial (LA) reservoir strain (LASr). A weak, non-significant positive trend was observed (r = 0.257, p = 0.142), which is likely attributable to the small sample size and should not be interpreted as a physiological relationship.

To further explore the clinical correlations of atrial dysfunction, patients were stratified based on LA reservoir strain less than 40% (n = 21) versus equal or greater than 40% (n = 13). Children with reduced strain had significantly larger LA volumes as measure by both four-chamber Z-score (p = 0.011) and biplane method (p = 0.029). Interestingly, systolic blood pressure was higher in those with preserved LA strain (p = 0.047), while diastolic pressure, ejection fraction, and GLS did not differ between groups (Fig 4).

Stage 1 hypertension was associated with significantly lower LA reservoir strain (*p* = 0.01) and larger LA volume (*p* = 0.02) compared to normotensive peers. No significant sex-related differences were observed in LA strain or volume.

Patients evaluated at least 24 months after surgery exhibited a non-significant trend toward lower LA reservoir strain (36.2 ± 6.1% vs. 39.0 ± 5.8%, *p* = 0.08), though LA volumes were similar. Stratification by age showed that children aged 2 years or older had significantly higher indexed LA volumes (*p* < 0.01), but LA strain remained comparable across age groups.

To complement these categorical analyses, continuous variable correlations were also examined. LA reservoir strain was not significantly associated with age (*p* = 0.767), weight (*p* = 0.994), height (*p* = 0.976), body surface area (*p* = 0.688), or time since surgery (*p* = 0.714). There was no significant association between LASr and age at repair (p = 0.57). Similarly, no correlation was found with systolic or diastolic blood pressure, ejection fraction, GLS, or LA volume. Notably, patients with LA dilation based on Z-score had numerically higher LA strain (41.7 ± 4.3%) compared to those without dilation (36.8 ± 6.2%), though this difference did not reach statistical significance (*p* = 0.078).

### Relationship between LA strain and diastolic function

When LASr was compared across diastolic function categories based on Z-scores of transmitral flow (E and A waves) and tissue Doppler velocities (septal and lateral e’), no statistically significant differences were observed (all p > 0.05). Similarly, when LASr was dichotomized (<40% vs. ≥ 40%), the distribution of abnormal Z-scores for E, septal e’, and lateral e’ did not differ significantly between groups, except for a higher proportion of abnormal A-wave Z-scores in the higher LASr group (p = 0.042). Overall, these findings suggest that LASr was not strongly associated with conventional Doppler-based diastolic parameters in this cohort.

## Discussion

Subclinical left atrial dysfunction was frequently detected in children after surgical repair of coarctation of the aorta, even when anatomical and hemodynamic outcomes appeared satisfactory. Although left ventricular ejection fraction and global longitudinal strain were preserved in all patients, the majority showed statistically significantly reduced LA reservoir strain (LASr) and conduit strain (LAScd) compared to reference data from healthy pediatric populations. Our data revealed weak inverse correlations between LA strain and both LVEF and GLS. This supports the hypothesis that LA dysfunction may be an early and independent marker of myocardial impairment.

Compared with previous studies on repaired coarctation of the aorta, which have focused mainly on left ventricular systolic performance, conventional Doppler diastolic indices, or left atrial size alone, our study provides one of the few comprehensive pediatric assessments of left atrial mechanics using both reservoir and conduit strain. Prior pediatric reports have largely described volumetric remodeling or relied on adult cohorts, whereas systematic evaluation of left atrial functional mechanics in children after CoA repair remains limited. By integrating speckle tracking derived LASr and LAScd with volumetric indices, our study offers incremental insight into early atrial dysfunction that may precede overt structural remodeling and may not be detected by standard echocardiography.

These findings align with those of Labombarda et al. [12], who reported that approximately 41% of adult patients exhibited reduced LA strain despite preserved LVEF after coarctation repair. Impaired LA strain in that study was associated with stroke and arrhythmias. In pediatric populations, research on LA strain remains limited due to the lack of standardized reference values and the challenges of enrolling healthy children as controls. As a result, and due to the absence of an internal control group in our study, we used reference data from a large meta-analysis of over 1,400 healthy children [11] assessed using 2D speckle-tracking echocardiography with EchoPAC, the same software used in our study. While some variability in population and imaging protocols may exist, this technical consistency supports the validity of the comparison in the absence of local normative data.

Among the 34 cases analyzed, LA dilation was observed in 20–50% of patients, depending on the measurement method. However, LA strain reduction was a more consistent and prominent abnormality. The mean LASr was 37.6 ± 6.2%, significantly lower than the pediatric reference value of 47.3% (95% CI: 42.5–52.1%; p < 0.001). LAScd was also significantly reduced (23.4 ± 8.3% vs. 32.8%, p < 0.001), whereas contractile strain (LASct) did not differ significantly. These findings suggest that functional impairment predominantly affects the reservoir and conduit phases, both of which rely on LA compliance and elasticity. In contrast, active atrial contraction appears to be preserved. This may represent an early phase of subclinical LA dysfunction prior to detectable structural or hemodynamic changes.

We further examined the relationship between LA strain and conventional Doppler-based diastolic indices. When LASr was compared across categories defined by Z-scores of transmitral flow (E and A waves) and tissue Doppler velocities (septal and lateral e’), no consistent or statistically significant differences were observed. Similarly, stratifying patients by LASr (<40% vs. ≥ 40%) revealed comparable distributions of abnormal diastolic Z-scores, except for the A-wave, which likely reflects age-related variation rather than a clear pathophysiological pattern. These results indicate that, in our pediatric post-CoA cohort, LA strain abnormalities were not strongly predicted by standard Doppler diastolic parameters. This supports the concept that LASr and LAScd may capture aspects of atrial dysfunction that are not fully reflected by conventional diastolic measures, highlighting their potential complementary value in echocardiographic follow-up. However, we acknowledge that comprehensive multiparametric diastolic staging (including left atrial pressure estimation and exercise assessment) was beyond the scope of this cross-sectional study and should be addressed in future longitudinal investigations.

Additionally, we observed a mild positive trend between LASr and systolic blood pressure (SBP), particularly among children with borderline or stage 1 hypertension. This may reflect a compensatory phase in which the LA temporarily increases its reservoir function to maintain left ventricular filling, despite mildly elevated afterload. The trend was not statistically significant and involved only a small subgroup. Thus, it does not affect the overall finding of reduced LASr following CoA repair. This observation supports the hypothesis of a preclinical phase during which LASr shows a transient increase before declining with progressive dysfunction. However, this observation should be interpreted with caution. Given the small sample size and cross-sectional design, the positive association between systolic blood pressure and LASr may reflect measurement variability, age heterogeneity, or a statistical artifact rather than a true physiological relationship. Accordingly, this finding should be considered exploratory and potentially non-physiologic, and no causal or compensatory mechanism can be inferred from the present data.

Further evidence from other pediatric populations also supports the role of LASr as a sensitive and disease-specific functional biomarker. In children with chronic systemic hypertension, Kaplinski et al. [17] reported preserved LASr (37.8% vs. 38.0%; p = 0.735), suggesting LA strain may remain intact during early adaptive phases of increased afterload. In contrast, Shakti et al. [18] found increased LASct and higher minimal LA volumes in children with moderate-to-severe congenital aortic stenosis, indicating compensatory atrial contractility in response to pressure overload. In another context, Doan et al. [19] observed decreased LASr during dialysis sessions in pediatric patients with end-stage renal disease, independent of changes in blood pressure or fluid volume. These findings collectively reinforce the notion that LASr is an early marker of atrial dysfunction. Moreover, it may serve as a pathophysiologically specific indicator across various hemodynamic conditions, including in the post-operative coarctation.

In parallel with these functional findings, LA strain and LA volume reflect complementary but distinct aspects of left atrial remodeling. Strain primarily represents atrial functional mechanics, whereas volume reflects structural changes that tend to occur later in the disease process. In our cohort, reduced LASr and LAScd were frequently observed even in children without marked LA enlargement, suggesting that functional impairment may precede overt volumetric remodeling. Therefore, LA strain may provide incremental information beyond conventional volumetric assessment in the mid-term follow-up after CoA repair.

It is important to note that all LA strain and volumetric measurements in this study were obtained using pediatric-optimized acquisition settings and age-appropriate normalization. Frame rates, imaging depth, and sector width were adjusted to account for smaller cardiac dimensions and higher heart rates in children, and volumes were indexed to body surface area using the Haycock formula. Moreover, LASr values were compared with a large pediatric reference dataset analyzed on the same EchoPAC platform, thereby enhancing the methodological consistency of our comparisons despite the absence of a local control group. Although we compared our findings with a large published pediatric reference dataset, we acknowledge substantial heterogeneity across pediatric strain studies, including differences in ultrasound equipment, software versions, imaging protocols, age distribution, hemodynamic status, and demographic characteristics. The absence of a contemporaneous local control group may influence both type I and type II error; therefore, comparisons with external reference values should be interpreted with caution and our findings should be considered hypothesis-generating rather than definitive.In addition, we did not perform three-dimensional (3D) LA strain or 3D volumetric analysis; future studies using 3D techniques may provide a more comprehensive assessment of atrial mechanics.

To better interpret these findings,the simultaneous reduction in LASr and LAScd likely reflects a functional interaction between left atrial (LA) mechanics and left ventricular (LV) diastolic properties. LASr represents the ability of the left atrium to accommodate pulmonary venous return during ventricular systole, whereas LAScd reflects passive emptying of the atrium during early diastole. Both phases are influenced, at least in part, by ventricular relaxation and compliance. Although we did not directly measure left ventricular filling pressures, subtle alterations in LV relaxation after coarctation repair could plausibly contribute to reduced atrial reservoir expansion and, consequently, to diminished conduit function. Alternatively, primary atrial remodeling related to chronic preoperative pressure load may also play a role. Therefore, the observed reductions in LASr and LAScd likely result from a combination of ventricular and atrial factors rather than a single isolated mechanism. Further longitudinal studies are needed to clarify these relationships.

Regarding clinical factors, the median age at surgery was 7.5 months (IQR: 3.0–39.0), with approximately 35% undergoing repair after 2 years of age. Older surgical age may be associated with prolonged exposure to elevated afterload and increased risk of atrial remodeling. However, no significant differences in LASr or LAScd were observed according to age at surgery, although indexed LA volume was higher in these patients. This supports the idea that LA strain is a more stable and sensitive functional parameter than structural indices. The median time from surgery to follow-up was 19 months (IQR: 9.0–59.0), and no significant correlation was found between LASr and postoperative interval (p = 0.714), further suggesting that LA strain is a relatively stable marker in mid-term follow-up. Consistent with this observation, we found no significant association between LASr and age at repair (p = 0.57). Given the limited sample size, this lack of association should be interpreted cautiously and requires confirmation in larger cohorts. Nonetheless, our cross-sectional design limits interpretation of the temporal evolution of LA dysfunction or its association with long-term clinical outcomes.

Several limitations should be acknowledged. The cross-sectional design and small sample size limit our ability to assess progression over time or identify independent risk factors. We recognize that our sample size (n = 34) limits the stability of multivariable analyses. Accordingly, all regression analyses in this study should be considered exploratory and hypothesis-generating rather than confirmatory. The findings from these models may be influenced by small-sample effects, potential overfitting, and statistical variability, and therefore require validation in larger, prospective cohorts. However, a post hoc power analysis confirmed that the primary comparison of LASr between our cohort and reference data was statistically well-powered (estimated power ≈ 0.95), despite the modest sample size. The minimal detectable difference at 80% power was approximately 7.5%, supporting the robustness of the observed effect size.

Even so, other design limitations remained, particularly the lack of a local control group. Additionally, some families declined further follow-up after surgery due to the child’s stable condition, making patient retention and sample size expansion challenging. Nevertheless, inter-observer reproducibility of LASr in this study was excellent (ICC = 0.943), supporting its feasibility and reliability in clinical practice. Future multicenter longitudinal studies are needed to confirm these findings and to evaluate the prognostic significance of LA strain after CoA repair.

## Conclusion

Subclinical left atrial dysfunction is prevalent in children after anatomically and hemodynamically successful coarctation repair, even in the absence of left atrial enlargement or systolic impairment. Reservoir strain was notably reduced and served as the primary marker in this study, given its high reproducibility and stronger validation in the literature. In addition, conduit strain, which reflects the conduit phase of the cardiac cycle, also significantly decreased, suggesting possible impairment of early diastolic left ventricular filling. However, due to the limited sample size, this finding should be interpreted cautiously and is primarily hypothesis-generating for future studies. Incorporating left atrial strain into routine follow-up may aid in early detection of silent dysfunction, and regular blood pressure monitoring remains equally essential after coarctation repair.

## Supporting information

S1 Fig Bland–Altman plot showing intra-observer reproducibility of LA reservoir strain measurement. The plot presents the mean difference (bias) between two observers (TTMP and PNV) as 1.5%, with 95% limits of agreement ranging from –3.08% to +6.08%. Most data points fell within these limits, indicating good agreement.
(TIFF)

S1 Table Individual-level left atrial strain and clinical characteristics. (PDF)

S2 Table Conventional Doppler-derived diastolic indices and Z-score distribution in the repaired CoA. (PDF)
