# Supplementary figures and images for: Left atrial strain reveals subclinical dysfunction in children after successful coarctation repair
Source: PLoS One. 2026 Mar 11;21(3):e0344778. doi: 10.1371/journal.pone.0344778 (PMC12978438; doi:10.1371/journal.pone.0344778)

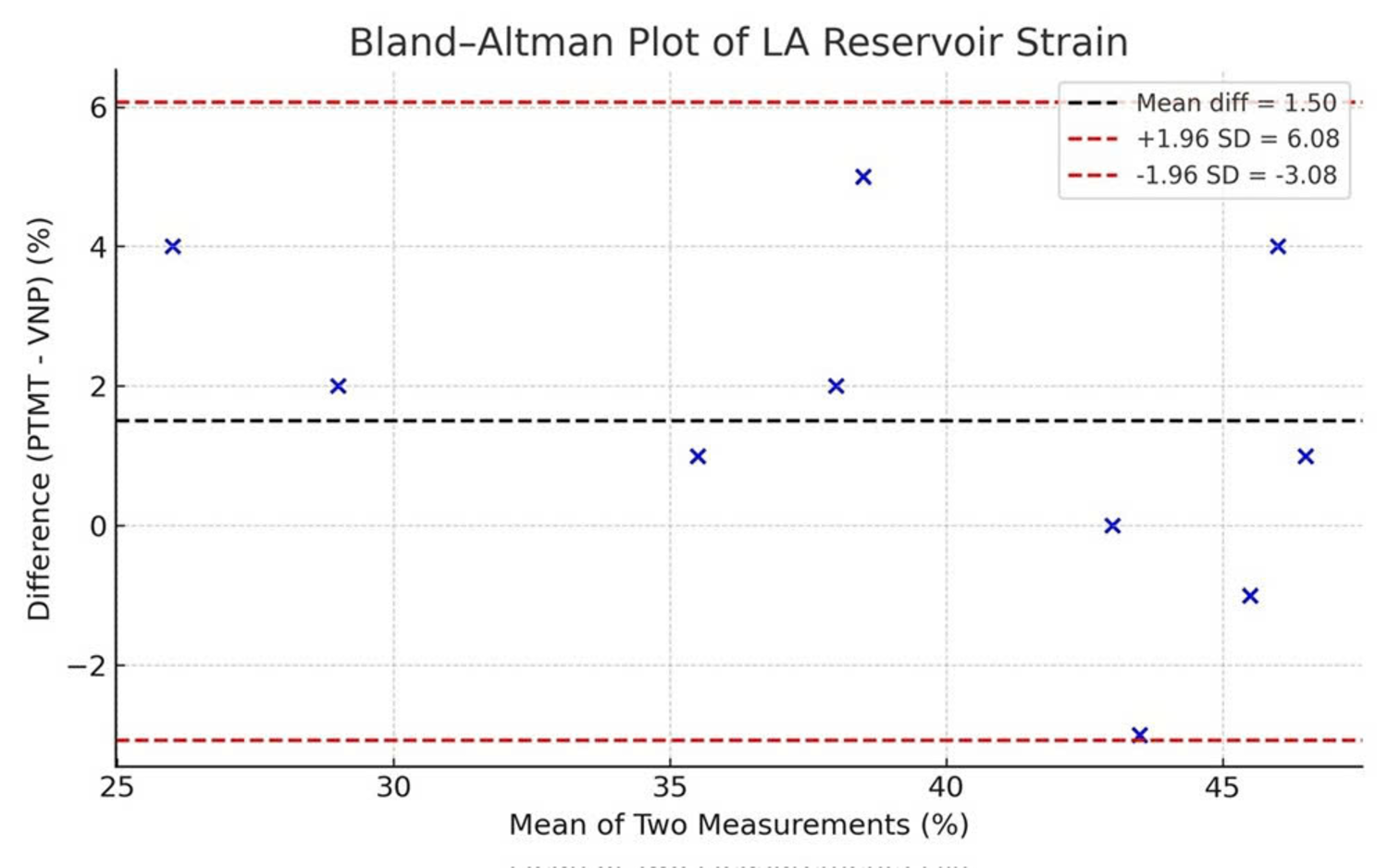

Supplement: S1 Fig — The plot presents the mean difference (bias) between two observers (TTMP and PNV) as 1.5%, with 95% limits of agreement ranging from –3.08% to +6.08%. Most data points fell within these limits, indicating good agreement. (TIFF) [file pone.0344778.s001.tiff]
