# Supplementary material for: Left atrial strain reveals subclinical dysfunction in children after successful coarctation repair
Source: PLoS One. 2026 Mar 11;21(3):e0344778. doi: 10.1371/journal.pone.0344778 (PMC12978438; doi:10.1371/journal.pone.0344778)
Supplement: S1 Table — (PDF) [file pone.0344778.s002.pdf]

| Case ID | Age (mo) | Sex | Height (cm) | Weight (kg) | BSA (m2) | LA Vol (Z-score) | LAVI (ml/m$^2$) | LA ESV (ml) | LA ESV Index (ml/m$^2$) | LA Strain Reservoir (%) | LA Strain Conduit (%) | LA Strain Contractile (%) | LVEF (%) | GLS (%) |
|---|---|---|---|---|---|---|---|---|---|---|---|---|---|---|
| Case 1 | 15 | F | 78.0 | 8.6 | 0.42 | -0.17 | 20.05 | 8.89 | 20.05 | 43 | 28 | 15 | 72.0 | -18.5 |
| Case 2 | 20 | M | 86.0 | 13.7 | 0.58 | 2.42 | 38.9868 | 20 | 38.9868 | 38 | 13 | 25 | 69.0 | -21.5 |
| Case 3 | 50 | M | 101.0 | 13.0 | 0.6 | 2.05 | 28.1296 | 20.12 | 28.1296 | 42 | 26 | 16 | 73.0 | -20.0 |
| Case 4 | 50 | M | 110.0 | 24.2 | 0.84 | 0.28 | 31.3909 | 26.79 | 31.3909 | 35 | 24 | 11 | 79.0 | -23.1 |
| Case 5 | 19 | F | 78.0 | 8.0 | 0.41 | -0.28 | 19.7379 | 8.22 | 19.7379 | 28 | 17 | 11 | 66.6 | -18.6 |
| Case 6 | 16 | F | 80.0 | 8.3 | 0.43 | -0.38 | 20.5337 | 8.44 | 20.5337 | 37 | 24 | 13 | 70.0 | -16.7 |
| Case 7 | 8 | F | 67.0 | 7.3 | 0.37 | 1.28 | 24.3568 | 9.0 | 24.3568 | 39 | 28 | 11 | 78.0 | -21.6 |
| Case 8 | 86 | M | 120.0 | 19.0 | 0.81 | 0.34 | 27.3888 | 23.5 | 27.3888 | 38 | 24 | 14 | 68.0 | -20.6 |
| Case 9 | 30 | M | 89.0 | 9.8 | 0.49 | 1.57 | 30.3231 | 14.0 | 30.3231 | 36 | 24 | 13 | 68.0 | -18.0 |
| Case 10 | 19 | M | 75.0 | 7.9 | 0.4 | 2.05 | 24.074 | 11.36 | 24.074 | 37 | 34 | 3 | 71.0 | -16.5 |
| Case 11 | 81 | F | 119.0 | 22.8 | 0.87 | 0.81 | 29.546 | 29.12 | 29.546 | 41 | 35 | 8 | 67.0 | -20.8 |
| Case 12 | 88 | M | 115.0 | 18.6 | 0.78 | -0.88 | 26.4935 | 18.0 | 26.4935 | 30 | 22 | 8 | 68.0 | -20.4 |
| Case 13 | 119 | M | 132.0 | 25.0 | 0.95 | -2.7 | 24.371 | 15.0 | 24.371 | 30 | 20 | 10 | 82.0 | -19.3 |
| Case 14 | 17 | M | 80.0 | 10.6 | 0.49 | 2.1 | 31.6218 | 15.0 | 31.6218 | 43 | 27 | 16 | 78.0 | -19.6 |
| Case 15 | 61 | M | 123.0 | 27.0 | 0.95 | 1.1 | 32.1565 | 35.5 | 32.1565 | 48 | 31 | 18 | 74.0 | -21.1 |

| | | | | | | | | | | | | | | |
|---|---|---|---|---|---|---|---|---|---|---|---|---|---|---|
| Case 16 | 13 | M | 98.0 | 9.2 | 0.42 | -2.0 | 12.7105 | 7.15 | 12.7105 | 39 | 32 | 7 | 78.0 | -20.0 |
| Case 17 | 19 | M | 80.0 | 9.8 | 0.45 | -0.49 | 21.2534 | 9.44 | 21.2534 | 45 | 1 | 44 | 68.0 | -17.5 |
| Case 18 | 42 | F | 90.0 | 11.0 | 0.52 | 1.67 | 25.5925 | 15.66 | 25.5925 | 30 | 20 | 10 | 76.0 | -22.0 |
| Case 19 | 104 | M | 130.0 | 23.0 | 0.93 | -1.9 | 23.7231 | 18.0 | 23.7231 | 47 | 29 | 18 | 66.0 | -22.5 |
| Case 20 | 11 | M | 70.5 | 8.9 | 0.4 | 0.73 | 22.8908 | 10.0 | 22.8908 | 39 | 21 | 19 | 65.0 | -18.3 |
| Case 21 | 55 | F | 90.0 | 11.5 | 0.53 | 0.02 | 24.9075 | 12.6 | 24.9075 | 44 | 30 | 14 | 74.0 | -18.0 |
| Case 22 | 24 | M | 74.0 | 9.5 | 0.42 | 1.42 | 28.6841 | 12.0 | 28.6841 | 37 | 1 | 37 | 69.0 | -18.5 |
| Case 23 | 131 | M | 120.0 | 22.0 | 0.86 | 0.02 | 27.1106 | 25.0 | 27.1106 | 28 | 16 | 12 | 79.0 | -18.4 |
| Case 24 | 44 | F | 95.0 | 13.5 | 0.59 | 1.12 | 28.3003 | 17.6 | 28.3003 | 44 | 33 | 11 | 71.0 | -21.6 |
| Case 25 | 66 | M | 100.0 | 13.6 | 0.61 | -1.19 | 39.7061 | 12.09 | 39.7061 | 33 | 16 | 17 | 75.0 | -20.9 |
| Case 26 | 196 | M | 166.0 | 49.0 | 1.53 | 2.46 | 60.9366 | 56.0 | 60.9366 | 49 | 38 | 11 | 71.0 | -20.1 |
| Case 27 | 101 | M | 143.0 | 49.0 | 1.37 | -1.09 | 29.2634 | 31.0 | 29.2634 | 30 | 21 | 9 | 77.0 | -18.2 |
| Case 28 | 98 | M | 116.0 | 24.0 | 0.87 | 0.84 | 46.704 | 30.0 | 46.704 | 44 | 22 | 22 | 68.0 | -20.6 |
| Case 29 | 102 | M | 128.0 | 22.0 | 0.9 | -0.57 | 43.3328 | 23.28 | 43.3328 | 33 | 22 | 11 | 65.0 | -20.3 |
| Case 30 | 152 | F | 150.0 | 37.0 | 1.26 | 1.08 | 52.1198 | 38.3 | 52.1198 | 28 | 15 | 13 | 64.0 | -19.4 |
| Case 31 | 8 | F | 65.0 | 6.2 | 0.32 | -0.31 | 29.6662 | 6.0 | 29.6662 | 29 | 22 | 7 | 75.0 | -17.7 |
| Case 32 | 129 | M | 140.0 | 31.0 | 1.11 | 5.5 | 90.4684 | 54.0 | 90.4684 | 41 | 33 | 8 | 73.0 | -21.4 |

| | | | | | | | | | | | | | | |
|---|---|---|---|---|---|---|---|---|---|---|---|---|---|---|
| Case 33 | 20 | M | 85.0 | 11.7 | 0.51 | -1.1 | 33.6748 | 9.95 | 33.6748 | 40 | 22 | 19 | 66.0 | -19.4 |
| Case 34 | 114 | M | 134.0 | 34.0 | 1.12 | -0.96 | 27.7675 | 15.0 | 27.7675 | 35 | 25 | 10 | 69.0 | -16.6 |
