# Supplementary material for: Left atrial strain reveals subclinical dysfunction in children after successful coarctation repair
Source: PLoS One. 2026 Mar 11;21(3):e0344778. doi: 10.1371/journal.pone.0344778 (PMC12978438; doi:10.1371/journal.pone.0344778)
Supplement: S2 Table — (PDF) [file pone.0344778.s003.pdf]

Supplementary Table S3. Conventional Doppler-derived diastolic indices and Z-score distribution in the repaired CoA.

Variables	Total (n=34)
**E ****	115.09 ± 21.14 (74.00 – 176.00)
**Z-Score**	
Z ≤ -2	0
-2 < Z < +2	23 (67.65%)
Z > +2	11 (32.35%)
**A ***	70.00 (62.00 – 87.00)
**Z-Score**	
Z ≤ -2	0
-2 < Z < +2	18 (52.94%)
Z > +2	16 (47.06%)
**E/A ****	1.52 ± 0.35 (0.85 – 2.27)
**Z-Score**	
Z ≤ -2	0
-2 < Z < +2	34 (100%)
Z > +2	0
**E' sep****	9.76 ± 2.47 (6.00 – 15.00)
**Z-Score**	
Z ≤ -2	0
-2 < Z < +2	22 (64.71%)
Z > +2	12 (35.29%)
**E' lat ****	11.25 ± 2.73 (5.00 – 18.00)
**Z-Score**	
Z ≤ -2	0
-2 < Z < +2	8 (23.53%)
Z > +2	26 (76.47%)
**E/E' sep ***	11.30 (8.40 – 14.30)

Variables	Total (n=34)
**E/E' lat ***	10.70 (7.90 – 12.60)
**E/E' avg ****	11.26 ± 3.77 (5.50 – 22.30)

** Median (IQR)*

*** Mean ± SD (min – max)*
